# Synchronized Brain Interactions Associated with Memory and Decision-Making

**DOI:** 10.1371/journal.pbio.0030432

**Published:** 2005-11-15

Next time you lose your keys, you might consider the Clark's nutcracker. During the fall, this woodland resident collects over 30,000 seeds, buries them in discrete locations, then returns over the winter to retrieve its cache. This improbable behavior requires the coordinated activity of different brain structures to integrate spatial coordinates encoded in the hippocampus with memories of how to find the seed stash. As it turns out, food-storing birds have a significantly larger hippocampus—a brain region involved in spatial organization and memory—than nonhording species.

In the laboratory, rats learning maze tasks also rely on hippocampal spatial information, which the prefrontal cortex integrates with memory of the route, task rules, and other relevant cues to direct navigational decisions. How the brain coordinates this activity is an active area of research. When neuron populations fire in sync, they produce oscillations in brain wave patterns (measured as local field potentials) that operate at many different frequencies. Brain wave frequencies called theta rhythms, which are prevalent in the rat hippocampus, are associated with working memory and decision-making in both animals and humans. Theta rhythms—which oscillate at about eight cycles per second—appear to act like a metronome for individual neurons that “phase lock” their firing in time with the theta rhythm.

Whether the synchronized activity of neuron populations across different brain structures correlates with functions like decision-making, and whether phase-locking somehow coordinates these diverse structures has remained an open question. But now, by training rats on a spatial working memory task—navigating a maze to a food reward—Matthew Jones and Matthew Wilson demonstrate a clear correlation between coordinated hippocampal and prefrontal cortex activity and memory or decision-making processes. [Fig pbio-0030432-g001]

**Figure pbio-0030432-g001:**
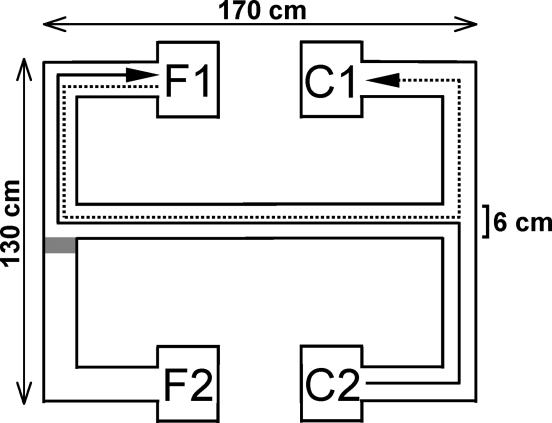
As rats ran through this maze, their brain activity was recorded to study the neural basis of memory and decision-making

Jones and Wilson first trained rats on a simple maze task. The maze was shaped like a stretched-out “H” (see diagram). Rats were trained to shuttle back and forth across the long central arm for about 20 trials per day. At one end of this central arm, a moveable barrier directed the rats to turn either left or right toward a chocolate reward. At the opposite end, rats encountered a free choice at the T-junction: the correct turn (leading to more chocolate) was contingent upon the direction in which they were previously directed by the barrier at the “forced turn” end of the maze. As rats ran toward this choice point, they therefore had to “hold in mind” both task rules and information about the preceding forced turn in order to decide upon the correct route. Like the nutcracker, flying from seed stash to seed stash in search of its food, the rats' performance presumably relies upon coordination of spatial information stored in the hippocampus with connected brain regions that guide behavior.

After rats had learned to correctly navigate the maze over 80% of the time for two straight days, they were outfitted with electrodes to search for neurons showing task-related activity. The authors recorded action potentials (activation signals) of groups of individual neurons, and local field potentials, from the medial prefrontal cortex (mPFC), which is associated with working memory and decision-making, and a hippocampal region called CA1 (named after the Egyptian god Ammon's horns, *cornu Ammonis* in Latin). It has been known since the early seventies that neurons in CA1 show spatially selective activity—that is, each neuron fires action potentials only in restricted regions of an animal's environment.

Firing rates of individual neurons in both CA1 and mPFC were indeed task-related: they distinguished between the directions of runs across the central arm, and between the different routes between reward points during the choice stages. The firing rates of CA1–mPFC neuron pairs coactivated during central arm crossings showed the highest correlations as rats ran toward the decision point. This correlated activity between the neuron pairs was significantly reduced when rats made mistakes and chose the wrong direction. Such synchronized activity, the authors explain, may represent the transfer of spatial information from the hippocampus to a working memory system in the mPFC.

Jones and Wilson go on to show that many CA1 and mPFC neurons were phase-locked to theta rhythms, with enhanced phase-locking during trials requiring working memory and decision-making. This effectively means that the firing of neurons in both structures was aligned to the same theta rhythm “metronome.” This, in turn, means that CA1–mPFC activities became correlated during distinct portions of the task. These correlations suggest that, as expected, the coordination and function of different brain regions depends on the task at hand. Additionally, this study shows that theta rhythms can be used as a reference against which to coordinate hippocampal and mPFC activity in accordance with behavioral demands of this maze task. Beyond shedding light on the neurobiology of behavior, these findings suggest that theta rhythms may contribute to diseases that involve disruptions in prefrontal cortex connectivity, such as schizophrenia—which, interestingly, can impair the spatial working memory of patients. —*Liza Gross*

